# Editorial: Theories of visual attention—linking cognition, neuropsychology, and neurophysiology

**DOI:** 10.3389/fpsyg.2015.00767

**Published:** 2015-06-12

**Authors:** Søren Kyllingsbæ, Signe Vangkilde, Claus Bundesen

**Affiliations:** Department of Psychology, Center for Visual Cognition, University of CopenhagenCopenhagen, Denmark

**Keywords:** neural, visual, attention, computational, model

The Neural Theory of Visual Attention of Bundesen et al. (2005) was proposed as a neural interpretation of Bundesen's (1990) theory of visual attention (TVA). In NTVA, visual attention operates via two mechanisms: by dynamic remapping of receptive fields of cortical cells such that more cells are devoted to behaviorally important objects than to less important ones (filtering) and by multiplicative scaling of the level of activation in cells coding for particular features (pigeonholing). NTVA accounts for a wide range of known attentional effects in human performance and a wide range of effects observed in firing rates of single cells in the primate visual system and thus provides a mathematical framework to unify the two fields of research.

In this Research Topic of Frontiers in Psychology, a host of new empirical findings from studies employing a theoretical and methodological framework based on NTVA are presented and discussed. The presented articles relate to the cognitive, neuropsychological, and neurocomputational levels of contemporary attention research and employ a variety of methods including behavioral testing, neuroimaging, and computational modeling.

In the first article of the Research Topic, Habekost (2015) offers a review of clinical TVA-based studies, in which the theoretical framework of TVA and NTVA is presented and discussed in relation to its clinical use. The review is followed by an article by Bogon et al. (2014), who present a TVA-based assessment of visual attention functions in developmental dyslexia. Following this, two papers on TVA-based measures of age-related effects and white matter brain microstructures are presented by Espeseth et al. (2014) and Wilms and Nielsen (2014). The fifth paper, by Nielsen and Wilms (2015), is also related to aging, but uses confirmatory factor analyses in Structural Equation Modeling in combination with TVA-based modeling. Next Bullock and Giesbrecht (2014) explore how acute exercise and aerobic fitness may influence selective attention during visual search. This paper is followed by a TVA-based study by Poth et al. (2014) combining a prospective memory task with traditional whole and partial report paradigms, thus studying effects of monitoring for visual events on distinct components of attention. Then Kyllingsbæk et al. (2014) present a study on automatic attraction of visual attention to supraletter features. In the final paper, Tsotsos and Kruijne (2014) propose an extension of their Selective Tuning model of attention, in which executive control over visual attention is implemented by Cognitive Programs. In the future, NTVA might also be extended with Cognitive Programs.

This collection of articles reflects the strong, continued interest in using a TVA-based framework for investigating and understanding visual attention in both healthy participants and patient groups, and the articles also provide important examples of how this may be done. If the reader should wish to delve into the latest theoretical developments of TVA and NTVA complementing the articles presented here, we further recommend the recent papers by Bundesen et al. (2014, 2015).

## Conflict of interest statement

The authors declare that the research was conducted in the absence of any commercial or financial relationships that could be construed as a potential conflict of interest.

## References

[B1] Bogon J. Finke K. Stenneken P. (2014). TVA-based assessment of visual attentional functions in developmental dyslexia. Front. Psychol. 5:1172. 10.3389/fpsyg.2014.01172 25360129 PMC4199262

[B2] Bullock T. Giesbrecht B. (2014). Acute exercise and aerobic fitness influence selective attention during visual search. Front. Psychol. 5:1290. 10.3389/fpsyg.2014.01290 25426094 PMC4227487

[B3] Bundesen C. (1990). A theory of visual attention. Psychol. Rev. 97, 523–547. 2247540 10.1037/0033-295x.97.4.523

[B4] Bundesen C. Habekost T. Kyllingsbæ S. (2005). A neural theory of visual attention: bridging cognition and neurophysiology. Psychol. Rev. 112, 291–328. 10.1037/0033-295X.112.2.291 15783288

[B5] Bundesen C. Vangkilde S. Habekost T. (2015). Components of visual bias: a multiplicative hypothesis. Ann. N. Y. Acad. Sci. 1339, 116–124. 10.1111/nyas.12665 25675882

[B6] Bundesen C. Vangkilde S. Petersen A. (2014). Recent developments in a computational theory of visual attention (TVA). Vision Res. [Epub ahead of print]. 10.1016/j.visres.2014.11.005 25458815

[B7] Espeseth T. Vangkilde S. A. Petersen A. Dyrholm M. Westlye L. T. (2014). TVA–based assessment of attentional capacities–associations with age and indices of brain white matter microstructure. Front. Psychol. 5:1177. 10.3389/fpsyg.2014.01177 25374549 PMC4204453

[B8] Habekost T. (2015). Clinical TVA-based studies: a general review. Front. Psychol. 6:290. 10.3389/fpsyg.2015.00290 25852607 PMC4364300

[B9] Kyllingsbæ S. Van Lommel S. Sørensen T. A. Bundesen C. (2014). Automatic attraction of visual attention by supraletter features of former target strings. Front. Psychol. 5:1383. 10.3389/fpsyg.2014.01383 25505892 PMC4245895

[B10] Nielsen S. Wilms I. L. (2015). Cognitive aging on latent constructs for visual processing capacity: a novel structural equation modeling framework with causal assumptions based on a theory of visual attention. Front. Psychol. 5:1596. 10.3389/fpsyg.2014.01596 25642206 PMC4295434

[B11] Poth C. H. Petersen A. Bundesen C. Schneider W. X. (2014). Effects of monitoring for visual events on distinct components of attention. Front. Psychol. 5:930. 10.3389/fpsyg.2014.00930 25191303 PMC4140074

[B12] Tsotsos J. K. Kruijne W. (2014). Cognitive programs: software for attention's executive. Front. Psychol. 5:1260. 10.3389/fpsyg.2014.01260 25505430 PMC4243492

[B13] Wilms I. L. Nielsen S. (2014). Normative perceptual estimates for 91 healthy subjects age 60–75: impact of age, education, employment, physical exercise, alcohol, and video gaming. Front. Psychol. 5:1137. 10.3389/fpsyg.2014.01137 25339932 PMC4187578

